# The Pathologic Role of Toll-Like Receptor 4 in Prostate Cancer

**DOI:** 10.3389/fimmu.2018.01188

**Published:** 2018-06-06

**Authors:** Tongwen Ou, Michael Lilly, Wei Jiang

**Affiliations:** ^1^Department of Urology, Xuanwu Hospital, Capital Medical University, Beijing, China; ^2^Division of Hematology and Oncology, Department of Medicine, Medical University of South Carolina, Charleston, SC, United States; ^3^Department of Microbiology and Immunology, Medical University of South Carolina, Charleston, SC, United States; ^4^Division of Infectious Diseases, Department of Medicine, Medical University of South Carolina, Charleston, SC, United States

**Keywords:** toll-like receptor 4, prostate cancer, inflammation, carcinogens, bacteria

## Abstract

Toll-like receptor (TLR) 4 is expressed on normal and malignant prostate epithelial cells. The TLR4 and its downstream signaling pathways mediate innate immune responses in the host against invading pathogens. However, multiple lines of evidence shows that TLR4 expression is increased in prostate tissues from prostate cancer patients, and altered TLR4 signals may promote cancer development, as well as antitumor effects. In this review, we have summarized key features of the TLR4 signaling pathway and its associated immune responses and focused on the pathologic role of TLR4 in prostate carcinogenesis and tumor progression.

## Introduction

Prostate cancer is one of the most common causes of morbidity and mortality in men. The prostate surrounds the upper part of the urethra. Retrograde translocation of bacteria from the urethra to the prostate may lead to chronic bacterial colonization of the prostate (1–4). Other possible points of entry of pathogenic organisms into the prostate may be through hematogenous and lymphatic dissemination from distant foci of infection (1–5). Indeed, infection and inflammation in the prostate are associated with increased risk of prostate cancer (6).

Infection and inflammation have been shown to associate with cancer development and progression. Colon cancer has a strong link with chronic inflammatory bowel diseases such as chronic ulcerative colitis and Crohn’s disease (7). Gastric cancer associates with chronic *Helicobacter pylori* infection and its mediated inflammation (8). Other examples include papillomavirus infection and cervical cancer, hepatitis virus infection and liver cancer, ovarian endometriosis and epithelial ovarian cancers, bronchitis and lung cancer, schistosomiasis and bladder cancer, pancreatitis and pancreatic cancer, cholecystitis, and gallbladder cancer (9–11). Similarly, prostatitis is linked to prostate cancer (6).

Mechanistic studies on the role of inflammation have been investigated in some cancers. For example, colorectal cancer cells express toll-like receptor (TLR) 4 and myeloid differentiation factor (MD) 2 complex, and lipopolysaccharide (LPS) stimulation activates phosphatidylinositol 3′-kinase/protein kinase B (AKT) signaling pathway and promotes downstream beta1 integrin function through the TLR4/MD2 complex, resulting in increased adhesiveness and metastatic capacity of colorectal cancer cells (12). Greten et al. found mouse colitis-associated pro-inflammatory cytokines play a role in colorectal cancer development (13). In addition, TLR4-signaling engagement promotes the adhesiveness and metastatic capacity of colorectal cancer (12). Furthermore, inflammation through TLR4 signaling promotes the development of immune suppressive microenvironment, such as recruitment myeloid-derived suppressor cells into local tumor environment, in breast, colon, renal cell, and pancreatic cancers (14–18). But no such evidence has been shown in prostate cancer. Thus, decreased antitumor immunity in the local microenvironment may account for another mechanism in tumor cell activation, proliferation, survival, invasion, and tumor metastasis. However, inflammation and prostate cancer have not been extensively studied, and the mechanisms involved are not clear.

In this review, we focus on the pathologic role of TLR4 and its effects in prostate cancer development and progression. This is important for therapeutic strategy targeting TLR signaling or responsiveness (e.g., inhibitors for specific pro-inflammatory cytokine) to prevent prostate cancer development and progression.

## Inflammation and Prostate Cancer

Inflammation is very common within the prostate, as there are about 10% male adults with prostatitis (19, 20). Several studies including a recent study found increased prostate cancer risk with a history of prostatitis and sexually transmitted diseases (relative risk = 1.30 and 1.43; 95% confidence interval: 1.10–1.54 and 1.07–1.91, respectively) (21). However, the mechanism of inflammation-mediated prostate cancer is unknown.

Proliferative inflammatory atrophy (PIA) is a precursor of prostatic intraepithelial neoplasia (PIN) and prostate cancer (22). Increased p53 immunostaining has been observed in PIA lesions, especially in the areas of acute inflammation (23). Studies from patients with bacterial prostatitis have revealed that several bacterial species induce inflammation and infect human prostate, and treatment with antibiotics results in a significant decrease in PSA levels, a marker for prostate cancer (24). *E. coli* and *Enterococcus* spp. are the most commonly bacteria found in bacterial prostatitis (25, 26). Upon stimulation, inflammatory cells infiltrate to the regions of prostatic atrophy and may contribute to the development of PIA, which contains regenerated atrophic epithelial cells in response to cell injury (22). Moreover, interaction and morphologic transitions between these lesions, PIA, PIN, and prostate cancer may contribute to cancer development and progression (23, 27). Notably, increased IL-6 and IL-6R expression has been observed in prostate cancer epithelial cells and high-grade PIN (28). Patients with metastatic prostate cancer have increased plasma IL-6 levels, which are correlated with PSA levels (29). These results suggest that inflammation such as bacterial infection, prostatitis, and pro-inflammatory cytokines (e.g., IL-6) may play a role in prostate cancer etiology and progression. Understanding the role of inflammation in prostate cancer development is critical as it has a great potential for therapeutic purposes.

## TLR4 Signaling Pathway

Toll-like receptors recognize invading pathogens, initiate innate immune responses, and potentiate adaptive immune responses (30–32). There are 10 TLR family members that differ in their expression patterns and agonist specificities in humans (33). Peripheral monocytes, macrophages, and normal epithelial cells from skin, digestive, reproductive, and respiratory organs have been shown to express TLR4 (32–34). The TLR4 ligand LPS is a major component of Gram-negative bacterial outer membranes (35). LPS triggers the TLR4 cell signaling pathway through initial binding to the LPS-binding protein and CD14, and subsequently to the TLR4/MD2 complex (36). Activation of the TLR4/MD2 complex results in conformational changes that enable downstream signal transduction (36).

Toll-like receptor 4 endogenous ligands [e.g., heat shock proteins (HSPs), fibrinogen, and high mobility group box 1 (HMGB1)] have been reported not only to initiate innate immune responses but also to function as danger-associated molecular patterns (37–39). Fibrinogen is mainly synthesized by hepatocytes; thrombin promotes fibrinogen to convert to fibrin and fibrinogen cleavage products, which function as TLR4 ligands and activate macrophages to produce inflammatory cytokines (40). Another endogenous TLR4 agonists, HSPs play a role in protein folding which directly bind to TLR4 on cell surface and activate dendritic cells through MAPK and NF-κB pathways, resulting in the production of pro-inflammatory cytokines (e.g., TNF-α, IL-1β, and IL-12p70) (41). Moreover, HMGB1 is a DNA-binding protein and plays a key role in the induction of inflammation after injury through TLR4/MD2 complex (42).

**Figure 1:**
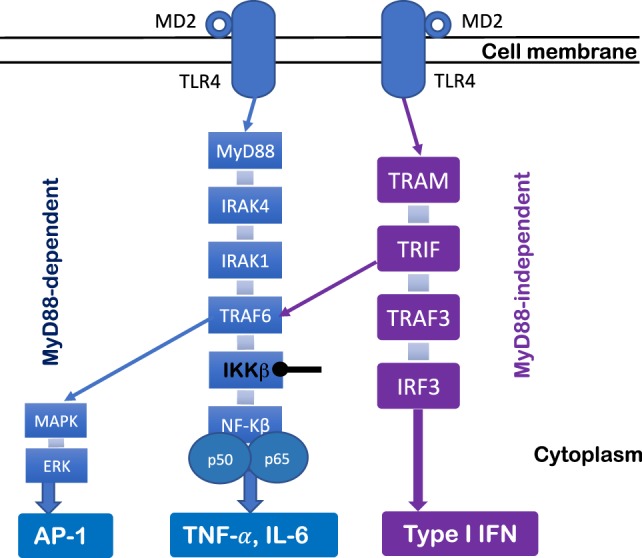
Toll-like receptor (TLR) 4 signaling pathway. TLR4 signaling pathway can be regulated by MyD88-dependent and MyD88-independent pathways. Upon the activation of MyD88, IL-1 receptor-associated kinase (IRAK) and tumor necrosis factor-receptor-associated factor 6 (TRAF6) are recruited to lead to degradation of IKKβ and disinhibition of NF-κB, which results in pro-inflammatory cytokine production (e.g., TNF-α and IL-6). The MyD88 pathway signals also activate MAKP and extracellular signal-regulated kinase (ERK) through TRAF6, which results in AP-1 activation. The MyD88-independent pathway is TRIF dependent. Upon the activation of TRIF-related adaptor molecule (TRAM)/TRIF, TRAFs are recruited, which leads to interferon regulatory factor 3 (IRF3) activation and type I IFN production.

The TLR4 signaling pathway has been extensively studied. Ligation of TLR4 complex triggers two distinct cell signaling pathways (Figure 1)—myeloid differentiation factor 88 (MyD88)-dependent pathway and MyD88-independent pathway. In the MyD88-dependent pathway, upon the activation of MyD88, IL-1 receptor-associated kinase and tumor necrosis factor-receptor-associated factor 6 (TRAF6) are recruited to induce activation of the inhibitor of κB (IκB) complex (IKK) and IκBα degradation, which results in the subsequent activation and nuclear translocation of NF-κB and pro-inflammatory cytokine production (e.g., TNF-α and IL-6) (43). Moreover, other cell signaling pathways also are involved in TLR4 signaling pathway [e.g., Jun N-terminal Kinase (JNK), p38, and extracellular signal-regulated kinase 1/2]. For example, the activation of TRAF6 further induces transforming growth factor-β-activated protein kinase 1 activation, resulting in the activation of members of the MAP kinase 3 (MKK3) and MKK6 which eventually activate pathways that phosphorylate JNK and p38 (43). Moreover, the MyD88-independent pathway is TIR-domain-containing adapter-inducing interferon-β (TRIF) dependent. Upon the activation of TRIF-related adaptor molecule/TRIF, TRAFs are recruited, and interferon regulatory factor 3 is activated, which leads to type I IFN production (Figure 1). Therefore, the activation of these cell signaling pathways after TLR4 engagement results in gene transcription and a cascade of inflammatory responses that initiate antimicrobial responses at the site of infection or inflammation (43).

## TLR4 Responses and Prostate Cancer

Toll-like receptor ligands have great potential as anticancer agents due to their adjuvant effects for adaptive immunity (44). Administration of TLR ligands in infrequent and high doses may act as immunologic adjuvants (44). On the other hand, low or physiologic doses of TLR agonists may mediate cancer development (5, 10), as evidenced by the use of TLR inhibitors to treat certain cancers (10, 45). TLR4 expression and its attendant chronic inflammation (e.g., IL-6) are associated with faster progression and poorer treatment outcomes in prostate cancer (5, 11, 44, 46, 47). Functional TLRs are expressed on a wide variety of cancer cells (10, 48) and may contribute to oncogenesis (10, 49–51). Chronic inflammation may result from infections and exposure to bacterial products (e.g., TLR agonists), and contribute to prostate carcinogenesis (11). However, in many patients, a source of chronic infection and inflammation is not apparent.

Toll-like receptor 4 stimulation through microbial molecules (e.g., LPS), as well as endogenous ligands (e.g., HSPs, fibrinogen, and HMGB1), induces pro-inflammatory cytokine production, such as IL-6, TGF-β1, TNF-α, IL-1β, inducible nitric oxide synthase, and antiapoptotic protein expression (40–42, 51). Notably, TLR4 is expressed on normal and malignant prostate epithelial cells (32, 47). Activation of TLR4 signaling in prostate cells initiates innate immune responses to invading pathogens. However, long-term activation of TLR4 cell signaling pathway in prostate epithelial cells may promote tumor cell activation, proliferation, survival, and tumor transformation (5, 47, 52). The microbial products lipoteichoic acid and LPS from oral pathogenic bacteria facilitate oncogenic herpesvirus infection in primary oral cells (53). In addition, TLR downstream cytokines IL-6, IL-8, and IL-10 mediate prostate cancer development and disease progression (54–57). Moreover, upon TLR4 ligand stimulation, prostate epithelial cells upregulate NF-κB, TGF-β1, and VEGF through increased TLR4 expression and induction of pro-inflammatory mediators (47, 58). Higher levels of some TLR4 downstream cytokines (e.g., IL-8) have been observed in prostate cancer tissues compared with control non-tumor tissues (59), suggesting that prostate cells experience persistent elevated inflammation presumably in response to bacterial products such as LPS as well as endogenous TLR4 ligands released from injured cells.

Immune suppressive microenvironment has been reported for other cancers except prostate cancer (14–18). We hypothesize that persistent bacterial LPS stimulation from bacterial colonization of the prostate may promote the development of immune suppressive microenvironment, including generation of T regulatory cells and tumor-associated macrophages and recruitment of myeloid-derived suppressor cells into local prostate tumor environment through TLR4 signaling. We hypothesize that these changes may result in decreased antitumor immunity and transformation from non-cancer prostate cells to prostate cancer cells. In addition, we hypothesize that long-term stimulation through TLR4 signaling in prostate cancer may lead to altered TLR4 responsiveness in tumor microenvironment. There is evidence on the role of increased plasma levels of fibrinogen and endogenous TLR4 ligands in breast cancer cell proliferation, survival, metastasis of cancer cells, and prognosis in patients (60, 61), however, their roles in prostate cancer are unclear. Whether systemic levels of TLR4 ligands (e.g., LPS, fibrinogen, and HSPs) are apparent in prostate cancer remains unknown. However, increased TLR4 expression and increased TLR4 responsiveness have been observed in prostate cancer cells (47, 58). Furthermore, TLR4 stimulation may have indirect effects on promoting prostate cancer development through reducing immune function in the tumor microenvironment (62, 63).

Knockout of TLR4 in PC3 prostate cancer cells decreases tumor cell migration and invasion (64). A TLR4 gene single nucleotide polymorphism may be associated with the risk of prostate cancer though results are not consistent (46, 65, 66). Peroxiredoxin-1, a TLR4 ligand, has been shown to interact with TLR4 to promote prostate tumor cell growth through chronic activation of cancer angiogenesis in a murine cancer model (67). Thus, TLR4-related innate immune activation and inflammation may play a role in the etiology and progression of prostate cancer (68, 69).

Toll-like receptor 4 may also contribute to the development of other cancers besides prostate cancer, including, but not limited to, liver cancer, reproductive organ cancer, pancreatic cancer, intestinal cancer, and skin cancer (45, 70, 71). For example, the knockdown of MyD88 results in a failure of ovarian cancer cell proliferation and cytokine production in response to LPS (50), suggesting that TLR4-MyD88 cell signaling contributes to epithelial ovarian cancer growth (50). Moreover, TLR4 ligands promote breast tumor progression *via* TLR4/NF-κB/STAT3 signaling (72). The anti-inflammatory compound andrographolide suppresses human colon cancer cell proliferation through the TLR4/NF-κB/matrix metalloproteinase-9 signaling pathway (73).

## Antibacterial and Anti-Inflammatory Treatment on Prostate Cancer

Recent advances in antibacterial and anti-inflammatory treatment on prostate cancer provide some information on the role of TLR activation in prostate cancer.

Gene silencing of TLR4 using small interfering RNA (siRNA) in PC3 cells significantly inhibits tumor cell migration and invasion, reduces cell viability, and mediates cell death (64). These effects are achieved by reducing expression (MyD88) or phosphorylation (TRIF, IRF-1) of TLR4 signaling pathway downstream-related molecules (64). Moreover, in a mouse prostate cancer model, TLR4 siRNA administration inhibits tumor growth and survival (64). These results suggest that TLR4 contributes to prostate cancer oncogenesis and progression (64). Furthermore, inhibition of LPS-mediated activation of TLR4 signaling pathway by selenium in human PCs cells results in decreased pro-inflammatory and likely anticancer activities (74).

Isothiocyanates, products from glucosinolates in plants, have anti-inflammatory, antiviral, and antibacterial properties. 3-Methylsulfinylpropyl isothiocyanate is shown to disrupt dimerization of the TLRs *via* covalent binding and thus has anti-inflammatory and TLR inhibitory activities (75). Moreover, phenethyl isothiocyanate inhibits TLR cell signaling pathway through interferon regulatory factor, which results in decreased IP-10 production in response to LPS (76). Furthermore, isothiocyanates induces cell apoptosis and cell cycle arrest, downregulates cell activation, and modulates epigenetic genes in PC3 cells, thus has anticancer activities (77–80).

## Antitumor Activity of TLR4

Mutation of TLR4 has been shown to result in susceptibility to infection by Gram-negative bacteria (81). TLR4 responses have been shown to confer antitumor activity. Recognition of TLR4 on antigen-presenting cells is able to enhance antigen-specific antitumor immunity (82, 83), therefore TLR4 agonists have been tested as antitumor therapy (84). We hypothesize that long-term low doses of microbial or endogenous TLR4 ligand simulation in local microenvironments (e.g., prostate) may promote cancer development; whereas one or two-time administration with high doses of TLR4 ligands (e.g., vaccine adjuvant) enhance antigen-specific antitumor immune responses. Further studies are needed to determine the mechanism involved for the contradictory effects of TLR4 on cancer.

## The Role of Other TLRs in Prostate Cancer

Toll-like receptor 9 has been shown to promote prostate cancer progression. Hossain et al. have shown that myeloid-derived suppressor cells express TLR9, accumulate in the circulation in patients with metastatic prostate cancer, and inhibit effector CD8+ T cell activity (85). Another study has determined that engraftment of TLR9-expressing prostate cells into mice results in the cross talk between TLR9 and leukemia inhibitory factor and promotes immunosuppressive activity through STATs and PMN-MDSCs (86). Moreover, TLR9 has been shown to be essential for propagation and self-renewal of prostate cancer cells through NF-κB/RELA and STAT3 pathways, and TLR9-positive tumors have a unique gene profile that associate with inflammation and stem cells (87). Importantly, TLR9 cytoplasmic immunostaining is positive in the majority of patients (66.7% strongly and 31.7% weakly positive) in prostate cancer cells (88). Although TLR9 expression is not associated with most markers of disease pathogenesis and progression (e.g., pT-class, Gleason score, and preoperative PSA level); it is associated with prostate cancer-specific progression-free survival in prostate cancer patients who have been treated by radical prostatectomy with curative intent (88). These results imply a pathologic role of TLR9 in prostate cancer progression. Targeting TLR9 signaling may provide therapeutic strategies for prostate cancer patients.

## Conclusion

In this review, we have summarized and discussed studies in TLR4 and prostate cancer and emphasized on the pathologic role of TLR4 in prostate cancer oncogenesis. This provides some insights about TLR4 inhibitors as potential prevention strategies for prostate cancer.

## Author Contributions

WJ wrote the first version of manuscript. ML and TO revised the manuscript.

## Conflict of Interest Statement

The authors declare that the research was conducted in the absence of any commercial or financial relationships that could be construed as a potential conflict of interest.
